# Feasibility and impact of implementing a private care system’s diabetes quality improvement intervention in the safety net: a cluster-randomized trial

**DOI:** 10.1186/s13012-015-0259-4

**Published:** 2015-06-10

**Authors:** Rachel Gold, Christine Nelson, Stuart Cowburn, Arwen Bunce, Celine Hollombe, James Davis, John Muench, Christian Hill, Meena Mital, Jon Puro, Nancy Perrin, Greg Nichols, Ann Turner, MaryBeth Mercer, Victoria Jaworski, Colleen Howard, Emma Abiles, Amit Shah, James Dudl, Wiley Chan, Jennifer DeVoe

**Affiliations:** Kaiser Permanente Northwest Center for Health Research, 3800 N. Interstate Avenue, Portland, OR 97211 USA; OCHIN, Inc., 1881 SW Naito Parkway, Portland, OR 97201 USA; Oregon Health Science University, 3181 S.W. Sam Jackson Park Rd., Portland, OR 97239 USA; Virginia Garcia Memorial Health Center, 2935 SW Cedar Hills Blvd., Beaverton, OR 97005 USA; Multnomah County Public Health Department, 426 SW Stark St, 8th Floor, Portland, OR 97204 USA; Kaiser Permanente Community Benefit, 6880 Paseo Laredo, La Jolla, CA 92037 USA; Kaiser Permanente Northwest Medical Group, 500 NE Multnomah Street, Suite 100, Portland, OR 97232 USA

**Keywords:** Community health centers, Quality improvement, Diabetes mellitus, Translational medical research

## Abstract

**Background:**

Integrated health care delivery systems devote considerable resources to developing quality improvement (QI) interventions. Clinics serving vulnerable populations rarely have the resources for such development but might benefit greatly from implementing approaches shown to be effective in other settings. Little trial-based research has assessed the feasibility and impact of such cross-setting translation and implementation in community health centers (CHCs). We hypothesized that it would be feasible to implement successful QI interventions from integrated care settings in CHCs and would positively impact the CHCs.

**Methods:**

We adapted Kaiser Permanente’s successful intervention, which targets guideline-based cardioprotective prescribing for patients with diabetes mellitus (DM), through an iterative, stakeholder-driven process. We then conducted a cluster-randomized pragmatic trial in 11 CHCs in a staggered process with six “early” CHCs implementing the intervention one year before five “‘late” CHCs. We measured monthly rates of patients with DM currently prescribed angiotensin converting enzyme (ACE)-inhibitors/statins, if clinically indicated. Through segmented regression analysis, we evaluated the intervention’s effects in June 2011–May 2013. Participants included ~6500 adult CHC patients with DM who were indicated for statins/ACE-inhibitors per national guidelines.

**Results:**

Implementation of the intervention in the CHCs was feasible, with setting-specific adaptations. One year post-implementation, in the early clinics, there were estimated relative increases in guideline-concordant prescribing of 37.6 % (95 % confidence interval (CI); 29.0–46.2 %) among patients indicated for both ACE-inhibitors and statins and 38.7 % (95 % CI; 23.2–54.2 %) among patients indicated for statins. No such increases were seen in the late (control) clinics in that period.

**Conclusions:**

To our knowledge, this was the first clinical trial testing the translation and implementation of a successful QI initiative from a private, integrated care setting into CHCs. This proved feasible and had significant impact but required considerable adaptation and implementation support. These results suggest the feasibility of adapting diverse strategies developed in integrated care settings for implementation in under-resourced clinics, with important implications for efficiently improving care quality in such settings.

**ClinicalTrials.gov:**

NCT02299791.

## Background

Integrated health care delivery systems such as Kaiser Permanente (KP) invest considerable resources in developing and implementing quality improvement (QI) initiatives and strategies for internal use. The community health centers (CHCs) constituting the health care safety net in the USA provide high-quality care to vulnerable (and growing) populations [1–3]; however, like most clinics serving low-income populations in the USA and internationally, these clinics have few resources for developing QI strategies comparable to those at large, integrated care systems. Patients and providers in clinics serving vulnerable populations would likely benefit from adopting approaches proven effective in better-resourced systems, but the feasibility and effectiveness of such cross-system adaptation and implementation have rarely been tested. Thus, we sought to determine whether and how interventions that work in private, integrated care settings can be translated into clinics with fewer resources, using CHCs in the USA as an example of such clinics.

We hypothesized that cross-setting translation is feasible and that adapting and implementing proven QI approaches could improve the care provided by under-resourced clinics without requiring them to develop native initiatives. We anticipated that this would involve substantially adapting potentially “translatable“ practices and interventions, due to the differences between private care settings and public clinics in terms of patient needs and vulnerability and system resources. We tested these hypotheses by adapting a diabetes QI initiative proven effective in KP, implementing it in 11 CHCs in a staggered-implementation, cluster-randomized pragmatic trial and measuring post-implementation impact in the CHCs.

To our knowledge, this is the first randomized trial of the feasibility and impact of translating a QI initiative developed and proven effective in an integrated care setting, for implementation in under-resourced clinics. Our overarching goal was to identify and resolve barriers to effectively implementing a successful, privately developed QI program into CHCs, to pave the way for future cross-setting dissemination of evidence-based programs into our nation’s safety net clinics and under-resourced clinics internationally. We report the results of this implementation trial.

### The A.L.L. Initiative intervention

Kaiser Permanente’s “A.L.L. Initiative” (Aspirin, Lovastatin (any statin), Lisinopril (any angiotensin converting enzyme-inhibitor (ACE) or angiotensin receptor blocker (ARB)); hereafter called “ALL”) is a system- and clinic-level QI intervention. Implemented throughout KP in 2003, ALL was designed to increase rates of patients with diabetes who are appropriately prescribed cardioprotective aspirin, statins, and ACE/ARBs. (Because the evidence for aspirin changed, we did not target it here.) At KP, ALL used electronic health record (EHR)-based tools coupled with top-down strategies to incentivize provider uptake. Its overarching strategies were to facilitate providers: (1) identifying patients with diabetes who were indicated for the ALL medications but not taking an indicated medication and (2) prescribing these medications. ALL was highly successful in KP: an internal study estimated a >60 % reduction in cardiovascular disease (CVD) events among targeted adults taking the ALL medications for 1–2 years [4, 5]. We selected ALL as a “test case” for studying cross-setting translational implementation based on its strong underlying evidence [4, 6–23], its alignment with national treatment guidelines, its impressive impact at KP, the simplicity of its strategies, and preliminary evidence that it could be adapted for the safety net [6].

### Adapting the intervention for implementation in the study sites

To adapt ALL for implementation in CHCs, we underwent an iterative, year-long process involving researchers, electronic health record (EHR) programmers, and CHC staff and providers, as previously described [24]. In brief, while the overall intervention aims and EHR tool functions remained the same as in KP’s implementation, each intervention component was customized to fit the CHCs’ needs and EHR capabilities. Each study CHC used the combination of components that best fit its existing workflows. KP’s top-down implementation strategies [25] were likewise adapted to emphasize practice facilitation [26–30], due to the CHCs’ different organizational structures and resources [31]. Table 1 summarizes the intervention components in both settings and how they were translated for implementation in the study CHCs. Details on the components of the implemented intervention were previously reported [24].

**Table 1:** Summary of ALL initiative when implemented in KP and as adapted for CHCs

	At KP	As adapted for and implemented in the study CHCs
Overarching strategies	Make it easier for providers to: (1) identify patients with diabetes who are indicated for an ALL medication(s), but have no active prescription for an indicated medication, and (2) prescribe these medications	
Target population		
Population “indicated” for ACE/ARBs and/or statins	Patients with diabetes at high risk of CVD (55-75, or comorbid CVD)	Any adult patients with diabetes (18-75)
Intervention components: Tools to expedite identifying patients indicated for but not prescribed ALL medication(s)		
Automated EHR point-of-care alerts “fire” at patient encounters if ALL medications indicated but not prescribed	Alerts added to existing, internally built “Patient Support Tool” which identifies myriad “care gaps” based on EHR data^1;2^	Alerts in the form of “Best Practice Alert” built into existing EHR functions; no other care gaps identified by this alert
Data registries enable searching provider/clinic panel for patients for whom ALL medications indicated but not prescribed	Integrated into existing panel tool; used to identify patients (i) on the day of a clinic visit, at the team “huddle,” and (ii) in targeted outreach efforts, in addition to other care gaps	Built as stand-alone ALL-specific rosters; provide similar functions as at KP (daily intake review; outreach)
Intervention components: Tools to expedite prescribing		
Order sets in EHR to make prescribing easier	Pre-programmed to expedite “one-click” prescribing for any indicated ALL medications (SmartSets)	Pre-programmed to facilitate prescribing by listing commonly prescribed dosages/medications
Intervention components: Tools to enhance patient adherence		
Patient education materials	EHR shortcuts that expedite providers’ ability to generate informational text about the medications in after-visit summaries	Similar EHR shortcuts; exam room poster about the ALL medications in English, Spanish, Russian; handouts to enhance adherence to prescribed medications in English, Spanish, Russian
Outreach to patients missing a prescription	Nurse, pharmacy case managers call patients to set up appointment to get prescription	At clinic discretion, used ALL registries to facilitate outreach to diabetic patients overdue for a visit
Compliance tracking	Nurse, pharmacy case managers call patients to remind them to refill their prescriptions	Not part of the CHCs’ intervention due to limited outreach capacity
Intervention components: Strategies to encourage provider uptake		
Communicate expectations related to intervention uptake	Top-down practice change directives	Presented as recommendations; staff input/feedback solicited
Orient staff to the evidence underlying the intervention	Champions presented at department meetings	Practice facilitators and/or clinician champions presented at clinic or team meetings (varied by organization)
Ongoing implementation support	Regional clinician champions responsible for multiple QI initiatives, including ALL	ALL-specific practice facilitators (clinic employees) provide on-the-ground support; clinician champions at each organization; research staff provides additional support
Performance tracking—providers	Monthly performance reports, posted publicly and tied to staff incentives	Monthly reports made available; emphasis, timing, and method of distribution varied by organization

## Methods

### Setting and data sources

The 11 study CHCs are ambulatory primary care clinics managed by three Federally Qualified Health Center (FQHC) systems in the Portland, Oregon metropolitan area. All are members of OCHIN, Inc., a non-profit organization that provides health information technology to safety net clinics [32–34]. The study CHCs vary in size and organizational structure: one is operated by a large academic medical center, though it is not located at that center; six by a county health department in urban locations; and four by a non-profit organization primarily serving suburban, Spanish-speaking populations. These clinics share a single EHR which is managed centrally at OCHIN, including regular data validation. EHR data for these analyses were extracted at OCHIN. Results from our extensive process evaluation will be reported in future manuscripts. [35]

### Design

In our staggered, cluster-randomized implementation strategy, six study clinics were randomized to implement the intervention in June 2011 (early implementation), and five 1 year later (late implementation). Cluster randomization was used because this is a clinic-level intervention with clinic-level outcomes. Randomization was matched on size of the clinics’ patient population and the FQHC system operating the clinic. The intervention’s effect was evaluated using an interrupted time-series design, analyzed with segmented regression models with calendar months as the unit of analysis [36].

### Main outcomes and measurements

Our outcome of interest was the proportion of patients with diabetes who were indicated for cardioprotective medication(s) (denominator) and had an “active” prescription for the indicated medication(s) (numerator), calculated monthly. We measured two clinic-level prescribing rates: (1) the proportion of patients indicated for ACE/ARB and statin who had an active prescription for both, and (2) the proportion indicated for statin only who had an active statin prescription.

#### Rate denominator

Each month’s rate denominator included clinic patients with diabetes who had a clinic encounter (in person or by telephone) in the last year and were indicated for one or both medications per current national care guidelines [37]. Patients were considered indicated for (a) an ACE/ ARB and a statin, if age 55–75 or age 18–54 with comorbid CVD; or for (b) a statin only, if age 18–54 with last low-density lipoprotein (LDL) >100. Patients currently pregnant or breastfeeding were excluded. Patients with a history of anaphylactic reaction to either medication were excluded from analyses involving that medication.

#### Rate numerator

Patients were considered to have an active prescription for an indicated medication class if they were prescribed that medication in the last year. Since the intervention targets provider prescribing rates, we consider this definition acceptable because it reflects the prescription data available to providers in the EHR.

### Statistical analyses

The early implementation effects on the two prescribing rates of interest were estimated using segmented regression models with two groups—the early implementation clinics were modeled as the intervention group and the late implementation clinics as the control group. We used data from 24 monthly intervals: 12 in the pre-intervention period (June 2010–May 2011) and 12 in the post-intervention period (June 2011–May 2012). Models included a constant term, a term to model the slope of the pre-intervention linear trend, a variable to indicate study group (intervention vs. control), terms to estimate change in level from pre- to post-intervention and change in slope of the trend, and interaction terms (group by pre-intervention trend, group by change in level, and group by change in trend) to test for significantly greater improvement in prescribing rates in the early implementation clinics, compared to the controls. The change in level provides an estimate of the immediate effect of the intervention; the change in trend, an estimate of its effect across time post-implementation.

Analyses of late implementation effects involved the late implementation clinics only, with level and trend of the pre-intervention period serving as controls for the post-intervention period. These analyses included 36 monthly intervals: 24 in the pre-intervention period (June 2010–May 2012) and 12 in the post-intervention period (June 2012–May 2013). Models contained a constant term, a term for the slope of the pre-intervention linear trend, and terms estimating pre/post-intervention changes in level and trend of outcome rates.

Preliminary qualitative analyses indicated that providers modified ALL medication prescribing based on patient age and gender. (Because of the teratogenic risks, some providers were wary of prescribing to younger females, and others were reluctant to prescribe statins to younger patients in general; details of these qualitative results pending in future papers). To account for potential differences in prescribing rates based on distribution of these factors across clinic groups, we adjusted all models for gender (percentage of denominator who were female at each time point), age (percentage of denominator age 18–39 on the first day of each month), and their interaction. All regression models also included a first-order autoregressive term to control for serial autocorrelation [36]. All analyses reflect tests of statistical significance with a two-sided α of 0.05 and were conducted using SAS Enterprise Guide 6.1 (PROC AUTOREG; SAS Institute Inc., Cary, NC, USA).

## Results and discussion

### Results

#### Patient demographics

For illustrative purposes, Table 2 presents patient characteristics by clinic group in June 2010 (baseline), June 2011 (beginning of early implementation), and May 2012 (beginning of late implementation). At baseline, 1152 patients with diabetes were indicated for both a statin and an ACE/ARB in the early implementation clinics and 879 in the late implementation clinics (Table 2). Most were aged 55–75. About 47 % had prescriptions for both indicated medications. There were 494 patients indicated for a statin only in the early implementation clinics and 424 in the late implementation clinics, about 2/3 of whom were aged 40–54. About 56 % of early implementation clinic patients had an active statin prescription as indicated, as did about 52 % of late implementation clinic patients. All 11 clinics stayed in the study throughout the study period.

**Table 2:** Patient demographics by clinic group at selected time points in study period

	June 2010		June 2011		May 2012	
	Early clinics	Late clinics	Early clinics	Late clinics	Early clinics	Late clinics
	Indicated for ACE/ARB and statin					
Patients with DM, no.	1152	879	1446	1179	1599	1436
% with CVD, age 18–39 years	0.2	0.1	0.3	0.2	0.4	0.4
% with CVD, age 40–54 years	7.9	4.8	6.3	5.0	6.8	4.9
% age 55–75 years	91.9	95.1	93.4	94.8	92.9	94.7
Gender						
% Female	61.0	61.9	60.4	58.8	58.3	58.0
Medication						
% with active prescription for ACE/ARB, statin	47.9	47.1	49.9	45.4	62.3	47.0
	Indicated for statin only					
Patients with DM, no.	494	424	607	624	761	720
% without CVD, age 18–39 years, last LDL >=100	34.2	30.9	32.8	32.2	30.6	30.7
% without CVD, age 40–54 years, last LDL >=100	65.8	69.1	67.2	67.8	69.4	69.3
Gender						
% Female	63.4	57.3	61.8	58.3	61.4	57.9
Medication						
% with active prescription for statin	55.7	52.1	51.6	47.8	63.7	51.8

#### Impact of the early implementation

**Fig. 1:**
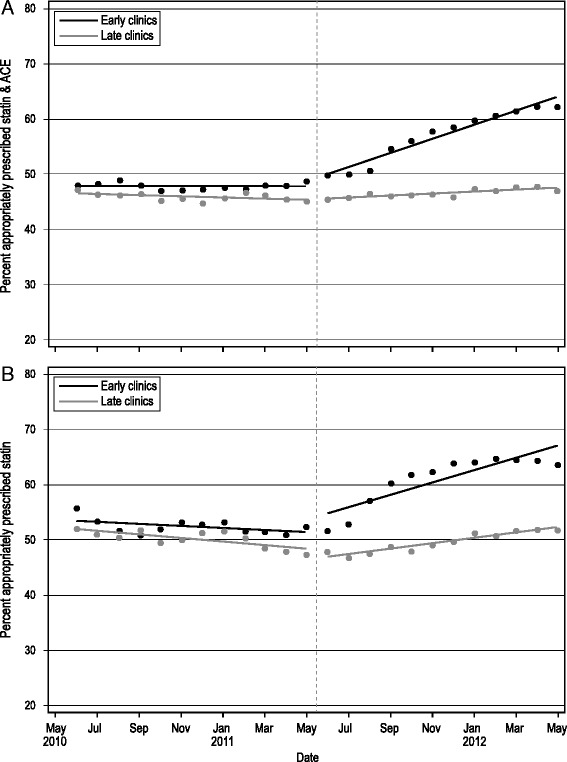
Effect of the early implementation of the ALL intervention. Time series of prescribing rates by month. **a** Statin and ACE among patients indicated for both drugs; **b** statins among patients indicated for statins only. *Dashed vertical line* indicates when early clinic implementation began (June 2011)

The effect of the intervention’s early implementation on guideline-concordant prescribing rates is shown in Fig. 1 and Table 3. Among patients indicated for both an ACE/ARB and statin (Fig. 1a), the pre-implementation trend in prescribing rates did not differ between the two groups (*p* = 0.744). The change in level, reflecting impact at the first time point post-implementation, was not significantly different between the groups (*p* = 0.685), but the groups did differ in the change in slope pre- versus post-implementation; the early implementation clinics had a significantly greater increase in this prescribing rate over time, compared to the late implementation clinics (*p* < 0.0001).

**Table 3:** Results of segmented regression analyses, early implementation effects (Controls = late implementation clinics)

	Estimate	Standard Error	*p* value
Percent actively prescribed statin and ACE/ARB, among patients indicated for both			
Difference in slope of the trend between control and intervention groups prior to the intervention	0.040	0.1208	0.744
Difference between control and intervention groups in change in level following the intervention	0.398	0.9742	0.685
Difference between control and intervention groups in change in slope of the trend from pre- to post-intervention	1.102	0.1706	<0.001
Percent actively prescribed statins, among patients indicated for a statin only			
Difference in slope of the trend between control and intervention groups prior to the intervention	0.103	0.241	0.673
Difference between control and intervention groups in change in level following the intervention	4.225	1.987	0.040
Difference between control and intervention groups in change in slope of the trend from pre- to post-intervention	0.491	0.318	0.131

Full models included a constant term, a term to model the pre-intervention linear trend slope, a variable for study group (intervention vs. control), terms to estimate change in level from pre- to post-intervention and change in slope of the trend, interaction terms (group by pre-intervention trend, group by change in level and group by change in trend), percent female, percent aged 18–39, gender by age interaction, and a first-order autoregressive parameter

Baseline trend lines were used to estimate an expected prescribing rate in the early implementation clinics after 1 year (by May 2012), if the intervention had not occurred. Using these methods, without the intervention, the prescribing rate in the early intervention clinics 12 months after implementation was estimated to be 45.8 %. With the intervention, the estimated prescribing rate was 63.0 %, representing a relative increase of 37.6 % (95 % CI; 29.0–46.2 %). The relative increase in the late implementation clinics in the same time period was estimated to be 8.4 % (95 % CI; −0.3–17.1 %).

Among patients indicated for statins only (Fig. 1b), prescribing trends declined slightly during the pre-intervention period and were not significantly different for the clinic groups in the pre-implementation period (*p* = 0.673). There was a significantly greater increase in prescribing rates immediately post-implementation in the early clinics, compared to the late clinics (*p* = 0.040). The slope of prescribing rates increased post-implementation, although the change in trend from pre- to post-implementation was not significantly different in early versus late clinics (*p* = 0.131). The estimated rate of statin prescribing in the early clinics without the intervention was 48.4 %. With the intervention, it was 67.2 %, a relative increase of 38.7 % (95 % CI; 23.2–54.2 %). The relative increase in the late clinics in the same time period was estimated to be 19.8 % (95 % CI; 2.7–36.9 %).

#### Impact of the late implementation

**Fig. 2:**
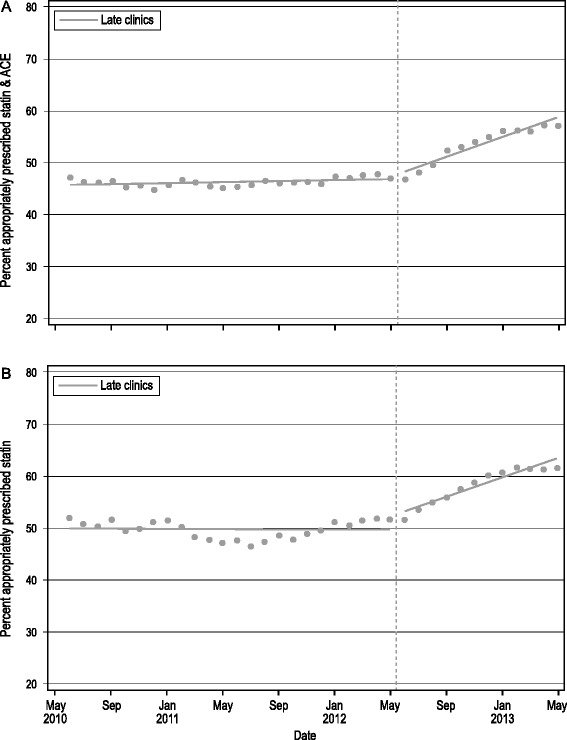
Effect of the late implementation of the ALL intervention. Time series of prescribing rates by month in late clinics only. **a** Statin and ACE among patients indicated for both drugs; **b** Statins only among patients indicated for statins only. *Dashed vertical line* indicates when late clinic implementation began (June 2012)

Guideline-concordant prescribing rates pre- and post- the late clinics’ implementation are displayed in Fig. 2. Among patients indicated for both ACE/ARB and statin (Fig. 2a), prescribing rates were flat in the pre-intervention period (slope = 0.0081, *p* = 0.9331). The slope of prescribing rates significantly increased post-implementation (change from pre- to post- implementation, 0.6552; *p* < 0.0001). Here, had the intervention not occurred, the estimated prescribing rate for ACE/ARB and statin would be 49.5 % at the end of the study period; with the intervention, the estimated prescribing rate was 57.2 %, a relative increase of 15.5 % (95 % CI; 9.0–22.1 %).

A similar response to the intervention was observed among patients indicated for statins only (Fig. 2b). The pre-intervention prescribing rate for statins was flat (slope = 0.009, *p* = 0.9377) and improved significantly following the intervention (slope change, 0.8246; *p* = 0.0011). If the intervention had not occurred, the statin prescribing rate at the end of the observation period was estimated to be 53.0 %. With the intervention, the estimated prescribing rate was 62.2 %, a relative increase of 17.3 % (95 % CI; 2.4–32.2 %).

### Discussion

There is a known need to expedite the dissemination of effective interventions across all care settings [38–40]. Doing so would facilitate the spread of proven interventions and QI approaches and reduce the need for care delivery systems to develop their own. Although this dissemination would be particularly useful to under-resourced clinics serving vulnerable populations in the USA and elsewhere, such clinics have historically been under-studied in dissemination and implementation science [41]. Instead, most previous QI efforts in CHCs and similar clinics were internally developed (a few exceptions cited here), and most cross-setting implementation research has focused on translation across similar care settings [28, 30, 41–48].

We believe this was the first clinical trial of the feasibility and impact of translating a QI intervention developed and shown effective in a private, integrated care setting, for implementation in under-resourced clinics. We showed that such translation and implementation is feasible but may require substantial adaptation to meet local needs and structures. In brief, we adapted the intervention components for implementation in the study clinics, as directed by an iterative process involving clinic staff. KP’s key strategies—making it easier to identify patients “missing” an indicated medication, and to prescribe that medication—remained the same; we adapted the specifics of how these strategies were implemented (including adapting the tools) and supported [24, 31].

Lessons learned about adapting QI interventions for implementation in under-resourced clinics include: (i) Consider the strategies used to support uptake of an adapted intervention [25]. Here, KP used top-down directives coupled with financial incentives; the CHCs used on-site facilitation. Though not a difference in the intervention itself, this could influence its uptake. (ii) Clinic cultures and leadership styles (e.g., degree to which top-down directives are issued and followed) can influence adoption of practice change initiatives, and should be considered when adapting such interventions. (iii) Though difficult and time-consuming, collaborative decision-making by clinic leaders (related to how to adapt the intervention) may be essential to eventual uptake. (iv) Ensure that the intervention aligns with the clinic’s standards of care; if possible, integrate it into the official standard of care. (v) Consider that low-income and otherwise vulnerable patients can face barriers to acting on medical recommendations—barriers not easily addressed through alerts and panel tools—and adapt as possible to address these barriers.

The demonstrated feasibility of effectively implementing a proven intervention in CHCs has important implications for safety net clinics in the USA and internationally. As such clinics rarely have the resources to develop “home-grown” interventions, implementing those shown effective elsewhere could yield important efficiencies in efforts to improve health care quality and outcomes. Such cross-setting translation should be considered a realistic means of helping lower-income clinics implement cutting-edge, effective QI strategies. Although implementing strategies from other settings requires adaptation to meet the clinics’ needs, it is likely more efficient than these clinics developing their own “from scratch”—an efficiency that could support dissemination of effective strategies across CHCs and similar care settings.

Comparing the impact of implementing ALL in the study CHCs versus KP is difficult for several reasons (some parallel study limitations, below). KP targeted patients with diabetes aged ≥55 and/or those with coronary artery disease at high risk for CVD, whereas in the study CHCs, the intervention targeted all adult patients with diabetes (an adaptation the CHCs requested). KP was able to measure uptake of ALL using dispense and refill data; we could only access prescribing data from OCHIN’s EHR, which until recently lacked medication dispensing data. Thus, this study would consider a patient’s care as appropriate even if the prescribed medication was never dispensed—a method of assessing guideline concordance that was less stringent by necessity. (Such primary non-adherence occurs in about 5 % of KP patients with diabetes [49] but was likely higher among uninsured CHC patients—preliminary qualitative analyses suggest that even small co-payments for prescriptions created an important barrier). KP started with considerably lower rates of concordance with targeted outcomes and at a time when there was less evidence supporting the medications’ effectiveness. When KP implemented ALL in 2003, about 33 % of their target population were appropriately taking the ALL medications; this rose to 52 % by 2005, a 58 % relative increase [4]. In the study CHCs, at baseline, about 48 % of patients indicated for an ACE/ARB and a statin were prescribed both. One year post-intervention, this increased to 62 % in the early clinics and 57 % in the late clinics. While we had thus limited ability to directly compare the impact of implementing ALL in KP versus the study CHCs, our results nevertheless show significant improvement in the CHCs.

The relative increases in guideline-concordant prescribing reported here range from 16–39 %. While these improvements are significant, the overall rates of appropriate prescribing ranged from 57–67 % by the end of the reporting period, meaning that 33–43 % of patients still did not receive prescriptions that were considered indicated. We believe this highlights limitations in the field’s current technical capacity and understanding of how to build effective EHR-based QI/decision support interventions. From a technical perspective, patients may be incorrectly identified as indicated for a given medication for myriad reasons that are potentially difficult to capture in an EHR-based algorithm, including: patient seeks pregnancy, complex concurrent medical conditions, potential medication interactions, and medication(s) prescribed elsewhere/entered in the chart in a way that does not trigger the algorithm. This illustrates the challenges inherent to striking a balance between false positives and false negatives when creating decision support algorithms in complex populations. This may also suggest that the implemented intervention was more successful than it appears, because some patients in the rate denominators could not be given the target medications. We plan to conduct further research on optimizing decision support tools adapted for safety net clinics.

#### Limitations

(1) We defined an active prescription as one issued in the last year, because the EHR contained data on medication prescriptions but not dispenses. We felt this acceptably reflected provider behaviors and information available to providers in the EHR, in the context of an intervention targeting provider prescribing behaviors. However, it does not describe rates of patients actually taking the medications; further research is needed to address this. (2) Some patients and providers likely “crossed over” from early to late clinics during the study year, even though the intervention components were only activated for staff at specified clinics per our randomization strategy; this is because a small percentage of CHC staff served patients at both early and late clinics, and the point-of-care alerts were seen by early CHC providers regardless of where they provided care. Though unavoidable in this pragmatic trial, any such contamination would underestimate intervention impacts, creating bias towards the null. (3) The technology and QI resources available needed to support such an intervention may not be available to other clinics, particularly those serving vulnerable populations.

#### Next steps/future research

This is the first of several planned papers on this study. We intend, in future manuscripts, to describe how the intervention’s impact sustained over time, its impact on health measures (blood pressure, lipid levels), and patient, provider, and system-level factors associated with use of the intervention components and implementation success. We are also developing a guide to implementing this intervention in CHCs. Our results indicate the need for research on the most effective methods for supporting future intervention implementation in under-resourced clinics in the USA and internationally and for improving the design of EHR-based decision support tools.

## Conclusions

This study adds substantially to our understanding of whether and how QI strategies proven effective in better-resourced care settings can be translated into under-resourced clinics. Such translation and implementation could potentially yield important benefits to clinics serving low-income populations by helping them keep up with the care delivery innovations that other settings develop. Developing strong practices for cross-setting implementation of effective innovations could substantially help safety net clinics benefit from tested interventions and processes and optimize health system performance [50].

## Abbreviations

ACE: angiotensin converting enzyme-inhibitor
ALL: A.L.L. Initiative (Aspirin, Lovastatin (any statin), Lisinopril)
ARB: angiotensin receptor blocker
CHCs: community health centers
CVD: cardiovascular disease
DM: diabetes mellitus
EHR: electronic health record
FQHC: Federally Qualified Health Center
KP: Kaiser Permanente
QI: quality improvement
